# Role of the RNA-directed DNA Methylation pathway in the regulation of maternal effects in *Arabidopsis thaliana* seed germination

**DOI:** 10.17912/micropub.biology.000504

**Published:** 2021-12-06

**Authors:** Ailén Authier, Pablo Cerdán, Gabriela A. Auge

**Affiliations:** 1 Fundación Instituto Leloir, Buenos Aires, Argentina; 2 Consejo Nacional de Investigaciones Científicas y Tecnológicas (CONICET), Argentina; 3 Instituto de Biociencias, Biotecnología y Biología Traslacional (iB3), Facultad de Ciencias Exactas y Naturales, Universidad de Buenos Aires, Buenos Aires, Argentina

## Abstract

The RNA-directed DNA Methylation pathway (RdDM) influences progeny seed responses to different maternal environments. However, its role in the regulation of early traits in response to non-stressful environmental cues across generations, which can potentially affect life cycle adjustment to seasonal changes, has not been explored in detail. Here we show that the RdDM pathway regulates overall germination but, in some instances, it does so depending on the early life maternal environment. Altogether, our results support that epigenetic memory (mediated by RdDM) is regulating intergenerational transmission of environmental information to affect the phenotypic expression of early traits.

**Figure 1. Effects of non-stressful temperature changes perceived by mother plants on germination responses of progeny seeds.**
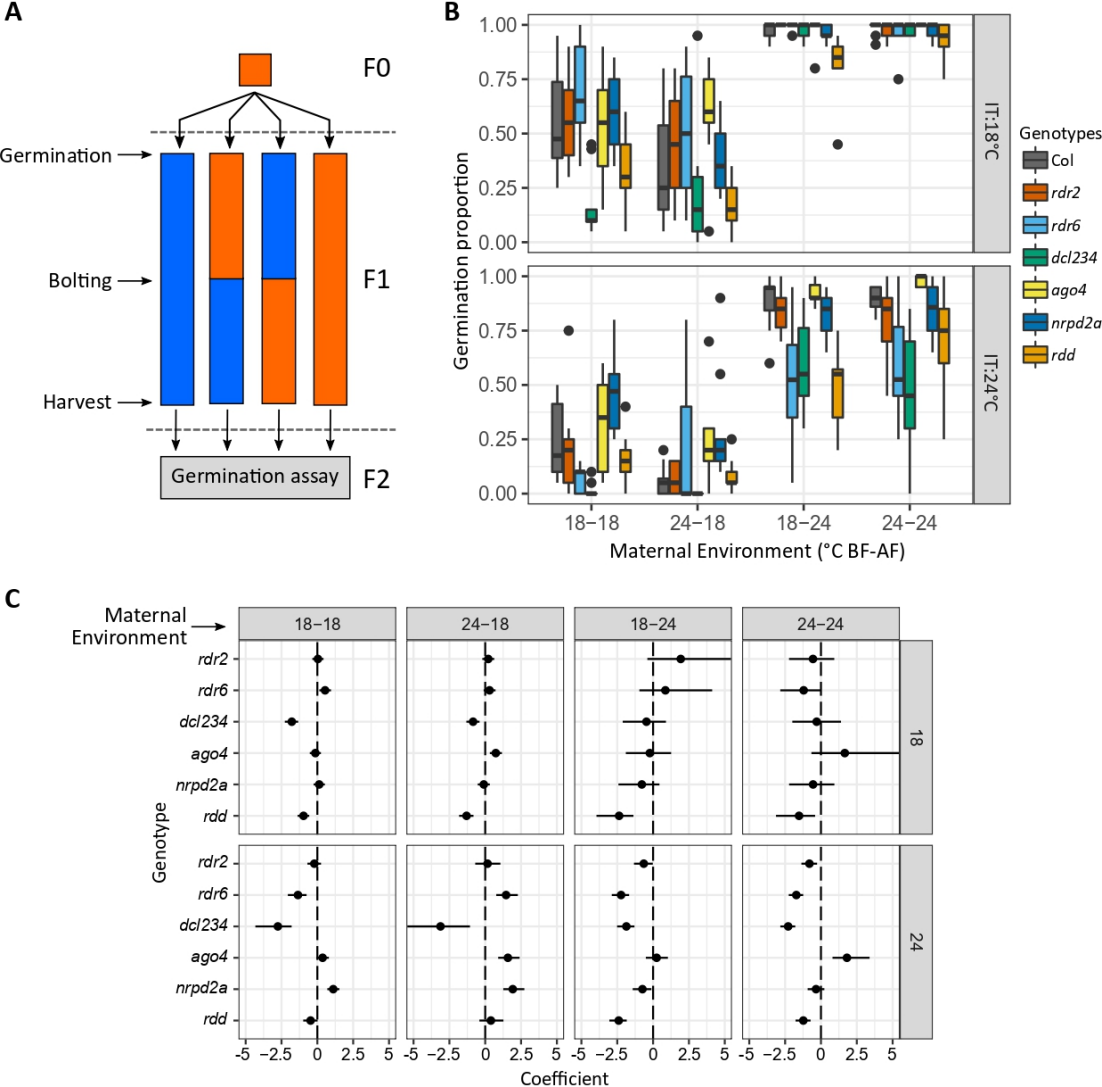
**A)** Experimental design: Plants of the maternal generation (F1) were grown at 18 (blue) or 24°C (orange) during their whole life cycles or changed to the contrasting environment after bolting. Seeds produced by mother plants grown in these four thermal environments were harvested and used to run a germination assay. **B)** Germination response of RdDM mutant seeds from mother plants grown in four thermal environments (x-axis) and incubated at 18 or 24°C (top and bottom panels, respectively). **C)** Direction and strength of the effect of each mutant on germination under each maternal treatment (columns) and incubation conditions (rows). Coefficients (x-axis) show the change in odds of germinating mutant seeds against the Columbia wild type, and their 95% confidence intervals. Positive values indicate mutant seeds germinated more than the wild type, while negative values indicate a decrease in germination for mutant seeds. Confidence intervals crossing ‘0’ (vertical dashed line) indicate there was no effect of the genotype.

## Description

The ability to process environmental information and adjust the phenotype in a way to face daily, seasonal and annual changes (phenotypic plasticity) is critical for survival, growth and reproduction ​of living organisms (Liancourt *et al.* 2013)​. Seeds respond to environmental cues experienced by mother plants, which can influence germination responses to and synchronization of the new generation of plants with optimal growth conditions and seasons (Donohue *et al.* 2010; Auge *et al.* 2017). Seeds from mother plants that experienced cold before flowering or during seed development and maturation show reduced germination—*i.e.*, dormancy is induced (Chen *et al.* 2014; Burghardt *et al.* 2016). Temperature effects on progeny germination are mediated by the RNA-directed DNA Methylation pathway (RdDM): cool temperatures experienced by mother plants after bolting induce methylation through RDR6 (RNA-dependent RNA POLYMERASE6) of an *ATHPOGON1* transposon located in the promoter region of the *ALLANTOINASE* (*ALN*) gene, a negative regulator of dormancy, reducing its expression and inducing seed dormancy (Iwasaki *et al.* 2019). RdDM is a pathway unique to plants that has been associated with multiple regulatory processes, including transposon silencing, responses to stresses, and development (Erdmann and Picard 2020). As RdDM influences the expression of transgenerational plasticity, we evaluated its role in the integration of the maternal life cycle environmental information and the expression of early developmental traits (germination) in seeds.

The seed environment showed a strong effect on germination: seeds incubated at 18ºC showed a higher germination proportion than those incubated at 24ºC for all genotypes and maternal treatments (Figure 1B). The maternal environment also showed a strong effect on germination as seeds from mother plants grown at warmer temperatures germinated more than those grown at cooler temperatures (Figure 1B). Interestingly, this effect was larger for seeds incubated at 18°C showing the maternal environmental history influences seed responses under certain environments (significant maternal treatment × incubation temperature, Table 2).

The timing of the environmental changes during the mother plant life cycle affected progeny germination, having a significant effect on the expression of genotypic variation (significant maternal treatment × genotype, Table 2). First, as expected, the environment after flowering showed a significant effect on progeny germination for both incubation temperatures and affected the expression of genotypic variability (18-18 vs. 18-24, and 24-18 vs 24-24; Table 3b; Figure 1C). Interestingly, the temperature before flowering affected progeny germination at both incubation temperatures as well: we observed significant interactions between the environment ‘before flowering’ and ‘genotype’ indicating that the maternal environment during vegetative growth, before seeds started developing, influenced the germination response of some genotypes (18-18 vs. 24-18, and 18-24 vs. 24-24; Table 3a; Figure 1C).

Given the strong genotypic effect, we analyzed the influence of the maternal environment for each genotype individually. Temperature before and after flowering experienced by the mother plants and the incubation temperature for each of the genotypes studied interacted in different ways to affect progeny germination for each genotype. Temperature before flowering influenced progeny germination and the expression of this effect depended on incubation temperature for Columbia, *rdr6* and *dcl234* seeds (significant ‘before flowering’ × ‘incubation temperature’ interaction, Table 4). This means that seeds responded to the maternal environment before flowering only when facing a certain temperature during imbibition; this could be due to conditions used in this study that can be masking other effects (*i.e.*, combination of cooler incubation and warmer maturation environments). On the other hand, for *nrpd2a* and *rdd* mutant seeds, ‘after flowering’ temperature influenced the expression of ‘before flowering’ and seed incubation temperature effects on progeny germination (significant ‘before flowering’ × ‘after flowering’, and ‘after flowering’ × ‘incubation temperature’ interactions, Table 4). For these genotypes, the maternal environment during seed maturation affected not only the expression of the early life cycle effects, but also the response of seeds to their own immediate environment. For *ago4* seeds, the effects of both before and after flowering maternal environments individually modified how the progeny responded to its immediate environment during imbibition (significant ‘before flowering’ × ‘incubation temperature’, and ‘after flowering’ × ‘incubation temperature’ interactions, Table 4). Finally, the response of *rdr2* seeds to the maternal environment showed a response influenced by the environments of both the mother plant and the seeds, showing a complex response to across and within-generation environmental cues (significant ‘before flowering’ × ‘after flowering’ × ‘incubation temperature’ interaction, Table 4).

Lastly, our results suggest that the response of progeny seeds to the thermal maternal environment is not mediated by the RdDM canonical pathway as the *rdr2* mutant did not show significant differences to the wild type in any of the conditions tested in this study (Table 5; Figures 1B and 1C). The RdDM non-canonical pathway mutants *rdr6* and *dcl234*, and the demethylases *rdd* mutant showed reduced germination, while *ago4* and *nrpd2a* germinated more than the wild type (Table 5, Figures 1B and 1C). Most effects were observed for seeds incubated at 24°C—incubation at 18°C could be masking genotype effects due to the inductive nature of that environment. A correct methylation homeostasis seems to be required for seeds to germinate and respond properly to their environment as *rdr6, dcl234* and *rdd* mutants showed an overall effect on germination. This could be caused by direct or indirect effects of RdDM on germination; however, we cannot discern between these possibilities with our experimental design. For other mutants, genotype effects on progeny germination showed specificity to the maternal environment: AGO4 would repress germination when mother plants are grown in warmer temperatures before flowering, while NRPD2A is likely repressing the response to cooler environments perceived after flowering (Figure 1B; Table 5). Whereas the response of *nrpd2a* mutant seeds is consistent with previous reports for RNA polymerase mutants (Iwasaki *et al.* 2019), the novel effect of *ago4* in impairing the response of the seeds to environmental cues experienced early in the mother plant life cycle needs to be studied further.

Altogether, our results suggest that integration of environmental information across generations through the RdDM pathway can potentially affect the expression of optimal phenotypes for early traits in response to previous generations’ environments and, in consequence, adaptive processes.

**TABLES**

**Table 1.** Information of the mutants used in this study.

**Table:** 

**Mutant**	**Mutant alleles**	**Gene ID**	**Ref.**	**Germplasm ID**	**Background**
Col-0			Lab stock		
*rdr2*	*rdr2-1*	AT4G11130	(Xie *et al.* 2004)	SAIL_1277H08	Col-0
*rdr6*	*rdr6-11*	AT3G49500	(Harmoko *et al.* 2013)	CS24285	Col
*dcl234*	*dcl2-1*	AT3G03300	(Deleris *et al.* 2006)	SALK_064627	Col-0
	*dcl3-1*	AT3G43920		SALK_005512	Col-0
	*dcl4-2*	AT5G20320		GABI_160G05	Col-0
*ago4*	*ago4-1*	AT2G27040	(Zilberman *et al.* 2003)	SALK_007523	Col-0 / L*er*
*nrpd2a*	*nrpd2a-2*	AT3G23780	(Onodera *et al.* 2005)	SALK_046208	Col-0
*rdd*	*ros1-3*	AT2G36490	(Penterman *et al.* 2007)	WiscDsLox469C11	Col-0 / Ws-0
	*dml2-1*	AT3G10010		WiscDsLoxHs088_08A	Col-0 / Ws-0
	*dml3-1*	AT4G34060		SAIL_563_E07	Col-0

**Table 2.** Interaction model between the variables ‘maternal treatment’ (Mat), ‘incubation temperature’ (IT) and ‘genotype’ (GT). Germination proportions were analyzed with logit-linked generalized linear models, and likelihood ratios were tested based on χ^2^. The triple interaction was dropped to increase power as it did not improve the model (based on Akaike Information Criterion, AIC). Table shows Chi-square (χ^2^), degrees of freedom (*df*) and *p*-values. Significance is shown with asterisks: ***, *p* < 0.001. Source of variation: ‘Mat’, maternal treatment; ‘IT’, incubation temperature; ‘GT’, genotype.

**Table:** 

**Source of variation**	**χ^2^**	***df***
Mat	880.28***	3
IT	102.12***	1
GT	156.00***	6
Mat × IT	35.66***	3
Mat × GT	234.99***	18
IT × GT	136.17***	6

**Table 3.** Effects of timing of maternal environmental changes (before and after flowering) on germination under each incubation temperature (‘IT’, columns). Maternal effects of temperature changes perceived by mother plants before (‘BF’) or after flowering (‘AF’) with the environment in the other life stage fixed, and their interactions with the variable ‘genotype’ (‘GT’). Environmental effects of **a)** before (24-24 vs. 18-24, and 24-18 vs. 18-18) or **b)** after flowering (24-24 vs. 24-18, and 18-24 vs. 18-28). Table shows LR Chi-square (χ^2^) and significant differences based on *p-*values: ***, *p* < 0.001.

**Table:** 

**Source of variation**	**χ^2^**	
	**IT: 18°C**	**IT: 24°C**
*a) Before flowering effects*		
	**24-24 vs 18-24**	
**BF**	0.53	0.05
**GT**	74.73***	208.37***
**BF** × **GT**	24.60***	36.53***
	**24-18 vs 18-18**	
**BF**	22.91***	33.54***
**GT**	145.62***	167.50***
**BF** × **GT**	36.52***	54.65***
*b) After flowering effects*		
	**24-24 vs 24-18**	
**AF**	270.85***	399.78***
**GT**	111.23***	134.61***
**AF** × **GT**	14.76***	105.11***
	**18-24 vs 18-18**	
**AF**	139.18***	227.06***
**GT**	145.62***	167.50***
**AF** × **GT**	46.06***	61.81***

**Table 4.** Maternal treatments and seed environment effects on germination responses for each genotype. Temperature effects perceived by mother plants before (‘BF’) or after flowering (‘AF’), and the interaction with the seed environment (‘IT’) tested for each genotype individually (columns). Table shows LR Chi-square values and significant differences based on *p-*values: ***, *p* < 0.001; **, *p* < 0.01; *, *p* < 0.05.

**Table:** 

	**Col**	***rdr2***	***rdr6***	***dcl234***	***ago4***	***nrpd2a***	***rdd***
BF	22.91***	3.82	14.62***	1.84	1.91	14.86***	13.75***
AF	139.18***	125.05***	78.99***	279.39***	117.92***	79.13***	97.20***
IT	54.49***	54.31***	161.76***	30.10***	17.85***	4.24*	13.75***
BF × AF	3.61	3.89*	1.76	0.05	4.28*	4.57*	24.11***
BF × IT	4.67*	4.38*	28.59***	5.14*	4.55*	0.01	0.19
AF × IT	0.48	9.75**	4.26*	0.55	0.27	6.94**	4.26*
BF × AF × IT	0.13	6.47*	0.11	1.85	0.78	0.27	0.36

**Table 5.** Genotypeeffects on the germination responses. Comparison between the Columbia wild type and RdDM pathway mutants within each maternal treatment (Mat: before and after flowering temperatures) and incubation temperature (IT). Table shows the LR Chi-square values and significant differences based on *p-*values: ***, *p* < 0.001; **, *p* < 0.01. See Figure 1c for effect sizes and direction of responses.

**Table:** 

	**IT: 18°C**				**IT: 24°C**			
**Mat →**	**18-18**	**24-18**	**18-24**	**24-24**	**18-18**	**24-18**	**18-24**	**24-24**
**Col-*rdr2***	0.04	6.51	5.50	0.60	0.80	0.24	1.96	8.83
**Col-*rdr6***	7.02	9.08	1.91	3.82	21.80***	23.36***	72.97***	54.10***
**Col-*dcl234***	68.32***	6.32	0.21	0.13	50.35***	14.99**	45.61***	93.17***
**Col-*ago4***	0.50	27.00***	0.00	3.35	2.78	28.73***	2.24	15.39**
**Col-*nrpd2a***	0.43	0.96	1.19	0.59	28.16***	46.71***	3.05	1.35
**Col-*rdd***	23.12***	18.67***	31.72***	7.73	3.67	1.28	87.56***	23.63***

## Methods

**Plant material and growth**

*Arabidopsis thaliana* plants of the Columbia accession (Col-0) and six null-mutants in the RNA-directed DNA methylation mechanism (RdDM, Table 1; *rdr2*,​ ​*rdr6,*​​ *dcl234,* ​*ago4-1*,​ ​*nrpd2a* and ​*rdd*)​ were used in this study (Table 1). The first generation of plants (F0) was initially grown to homogenize the environment in which seeds of the maternal generation (F1) were produced with the goal of removing any undesired intergenerational effects. F0 seeds were imbibed on plates with agar-agar 0.6% w/v (Plant Agar, Duchefa Biochemie, Cat no. P1001) for 4 days at 4°C in darkness. Seeds were transferred using a micropipette to 180 ml pots with a mix of 2:1 soil:perlite (Grow Mix Multipro, Agroquímica Larroca SRL., Argentina). Pots were immediately placed in a walk-in growth chamber where seeds were allowed to germinate at 24°C in a long day photoperiod (LD, 16h light / 8h darkness, white light sources were Neutral LED tubes 4100K, PAR ~130 μmol m^−2^ s^–1^). One week after germination, seedlings were thinned leaving one focal plant per pot. Plants were grown at 24°C and LD until harvest. Plants were watered regularly (2-3 times a week) and fertilized once with Red Hakaphos 0.1% w/v (Compo Agricultura, Argentina) immediately after bolting. Seeds were harvested when 2/3 of the fruits were dry and placed in an airtight container with silica-gel for 3 days. Seeds from 3 plants of the same genotype were pooled, stored in 1.5 ml tubes to generate the long-term F1 seed stock, and kept in a fridge until used for experiments. To explore the temperature effects on the expression of plasticity across generations, we grew plants at two temperatures that are known to have contrasting effects on *Arabidopsis* growth: 18 and 24°C. We either grew the plants at those temperatures during their whole life cycles (18-18 and 24-24), or they were moved to the contrasting temperature after flowering (18-24 and 24-18), obtaining a combination of four possible thermal environments (Figure 1A).

**F2 seeds germination assay**

To evaluate the maternal effects on germination and interaction with the seed’s environment, freshly harvested seeds (3 days after harvest) were imbibed in agar-agar plates (0.6% w/v) and immediately incubated in LD at 24 or 18°C. Twenty seeds per genotype, replicate, maternal treatment, and incubation condition were sowed per plate (504 plates total). Germination was counted daily during the first week of incubation and then every other day until seeds reached a germination *plateau* (no new germination in two consecutive counting days). A seed was considered germinated when radicle protrusion was observed. The final germination proportion was calculated as the number of germinated seeds divided by the total number of viable seeds per plate.

**Statistical analysis**

All statistical analyses were conducted using R v. 3.6.1 (R Core Team, 2020). To test for effects of the different temperature environments on F2 seed germination, we fit generalized linear models with logit link functions using ‘glm’, and then performed type-III likelihood ratio tests (based on Chi-square, χ^2^) using the ‘Anova’ function in the ‘car’ package (Fox and Weisberg 2010). As germination is a binomial trait expressed in form of proportions, we used a logit link function to run the tests. We first analyzed the effect of the whole life cycle environment, followed by an analysis of the environment effects before (BF) and after flowering (AF) and their interaction (BF × AF) to evaluate whether the timing of the changes in the environment had an influence on the germination response. We also analyzed genotype effects (mutants against the reference genotype, Col-0) within each combination of maternal treatments and incubation temperature. To analyze the strength and direction of genotype effects in each maternal environment and incubation conditions, we fitted biased reduced generalized linear models for each mutant against the wild type (‘brglm’ package; Kosmidis 2021) to show the change in odds (‘Coefficient’) on seed germination and 95% confidence intervals.

**Data availability**

https://osf.io/wmcah

## Reagents

Plant Agar, Duchefa Biochemie, Cat no. P1001
