# Erratum: Role of the RNA-directed DNA Methylation pathway in the regulation of maternal effects in *Arabidopsis thaliana* seed germination

**DOI:** 10.17912/micropub.biology.000527

**Published:** 2022-02-14

## Description

In the version of the microPublication originally published online, the link to extended data file was omitted from the original article, this has been corrected. A link to the extended data file <Authier et al_GerminationData_Metadata_Micropublication 2021.csv> has been attached to the original article.

A reference to the extended data file has also been added to the figure legend (1B and 1C).

